# Clinical Evidence of Magistral Preparations Based on Medicinal Cannabis

**DOI:** 10.3390/ph14020078

**Published:** 2021-01-21

**Authors:** Sara Arias, Marta Leon, Diego Jaimes, Rosa-Helena Bustos

**Affiliations:** 1 Evidence-Based Therapeutics Group, Clinical Pharmacology, Universidad de La Sabana, Chía 140013, Colombia; saraarvi@unisabana.edu.co (S.A.); diegojf@unisabana.edu.co (D.J.); 2 Pain and Palliative Group, Universidad de La Sabana, Chía 140013, Colombia; martha.leon@unisabana.edu.co

**Keywords:** magistral preparation, medicinal cannabis, anorexia, cachexia in HIV, post-chemotherapy nausea and vomiting, neuropathic pain in multiple sclerosis, pharmacology

## Abstract

Cannabis has been widely used as a medicinal plant for millennia; however, studies related to its main components were first conducted in 1960. Subsequently, laboratories have produced new components and structures related to its active biological properties. Countries that have approved the medicinal use of cannabis impose regulations that govern its clinical and scientific use. One means of administering medicinal cannabis is via a magistral preparation that must have a medical prescription and be prepared in an establishment that meets quality standards to ensure the quantities of its main components, such as tetrahydrocannabinol (THC) and cannabidiol (CBD). Furthermore, suppliers must have a clear indication of its use in the patient before prescription. This review shows the published evidence regarding the clinical use of medicinal cannabis magistral preparations in the management of post-chemotherapy nausea and vomiting, neuropathic pain in multiple sclerosis, and anorexia and cachexia in patients with HIV.

## 1. Introduction

The history of cannabis dates back to the year 4000 BC, with its first remains found in China. The first records of the use of cannabis are also found in a Chinese treaty, according to which, marijuana tea was used for the treatment of gout, rheumatism, malaria, and memory loss. However, the treaty warns of the main adverse effects, such as hallucinations and a feeling of “light body” [1]. Subsequently, its medicinal use spread through Asia, the Middle East, and Africa, where it was used for religious, social, and cultural purposes. These included the manufacture of ropes and health treatments, for which it showed benefits for use in certain refractory diseases as an alternative to conventional management [1].

This situation changed with the stigmatization of use at the end of the 19th century, when the epidemic of opioid abuse arose and legislation was introduced that regulated chemical substances related to psychoactive components, mainly as a control measure to address addiction. Therefore, in 1937, the Marijuana Tax Law was approved in the US, which made the non-medicinal use of cannabis illegal. In 1939, the Colombian government prohibited the cultivation of the plant and ordered the destruction of existing plantations [2].

Documents and evidence related to the use of medicinal cannabis in a clinical context are scarce. According to a review of the research, the National Academy of Sciences, Engineering, and Medicine concludes that there is conclusive or substantial evidence of the effect of cannabis on chronic pain, chemotherapy-induced nausea and vomiting, multiple sclerosis-associated spasticities, and refractory epilepsy. Moderate or low evidence indicates an effect on sleep disturbances (due to obstructive sleep apnea syndrome, fibromyalgia, chronic pain, and multiple sclerosis) [3,4,5], Hungtington’s disease, additions, glaucoma, Parkinson’s disease, Tourette syndrome, anxiety, cancer, Lennox–Gastaut syndrome, or Dravet syndrome [6,7,8,9].

One means of administering medical cannabis is via compounding formulas. According to the technical definitions of the US Pharmacopoeia, chapter 795 [10], and the European Pharmacopoeia monograph on “pharmaceutical preparations” [11], pharmaceutical preparations are classified as non-sterile preparations and defined as the preparation, mixture, assembly, alteration, packaging, and labelling of a drug, combined with formulated excipients and drug delivery device or devices, according to a medical prescription and for an individual patient. These must be preparations made by a pharmaceutical establishment created specifically to comply with a specific prescription for a patient with an indication. Furthermore, they must also have a prescription endorsed by a health professional and be prepared in facilities with good practice regulations.

In the context of medicinal cannabis in Colombia, under the regulations of Decree 613 of 2017 [12], these magistral preparations relate to the pharmaceutical form via which the drug can be dispensed to facilitate its administration, dosage, and release. These preparations must comply with all regulations applicable to pharmaceutical products, and the concentrations of tetrahydrocannabinol (THC) and cannabidiol (CBD) must be specified to determine the dosage [13,14]. Cannabis derivatives that are required as raw material for magistral preparations can only be provided by natural or legal persons that have a national license to manufacture cannabis derivatives [12,14,15,16,17,18,19].

Dispensing of these magistral preparations must include [18]: common and scientific name of the plant material (genus, species, variety, and author); specification of the part of the plant used; in the case of extracts and tinctures, the solvent used; the ratio between the weight of the material of the medicinal plant and the volume of the solvent; the content of active substances; the pharmaceutical form and route of administration; concentration of the final content of THC and CBD to determine the dosage; special instructions for storage, preparation, and administration; manufacture and expiration dates; and batch number [17,20].

The regulations in Colombia regarding the magistral formulation relate to the following: (i) The norms for the control, monitoring, and surveillance of the import, export, processing, synthesis, manufacture, distribution, dispensing, purchase, sale, destruction, and use of substances subjected to control, medicines, or any other product other than cannabis, its resin, extracts, and tinctures on the yellow list of narcotic drugs, and THC on the green list of psychotropic substances [21]. (ii) Regulation of the registration and licensing regime, control quality, and health surveillance regime for drugs, cosmetics, and pharmaceutical preparations based on natural resources that have traditionally been used empirically for therapeutic purposes [22]. (iii) A regulatory framework that allows safe and informed access to medical and scientific use of cannabis and its derivatives in Colombia [23]. (iv) Safe and informed access to the medical and scientific use of cannabis. Additionally, it is the first decree that discusses the magistral preparations and commercialization of products derived from cannabis [12]. (v) Licenses for the production and manufacture of cannabis derivatives [24]. Resolution 2892 of 2017 regulates the licenses for the production and manufacture of cannabis derivatives [24]. (vi) Updating of narcotic, psychotropic, and precursor drugs. Article 5 notes the classification of pharmaceutical products derived from cannabis, in which those of chemical synthesis and magistral preparations with an amount equal to or greater than 2 mg of THC are classified as special control drugs, whereas those with a lesser amount are considered non-audited products [25].

To understand the importance of the key characteristics of medical cannabis, its pharmacokinetics and pharmacodynamics are described in Table 1.

Concerning the pharmacodynamics of medicinal cannabis, the endocannabinoid system is responsible for maintaining body homeostasis by regulating temperature, sugar, pH, and the elimination of metabolites. Its activation can be conducted by endocannabinoids (anandamide and 2-arachidonylglycerol (2-AG)), by phytocannabinoids (plant derivatives: leaves, tinctures, and extracts with components of THC, CBD, CBN, etc.) and synthetic cannabinoids (products of pharmaceutical laboratories of THC analogues (Nabilone), THC/CBD extracts (nabiximols—Sativex^®^), synthetic THC (Dronabinol), and pure CBD extract (Epidolex). The above ligands can act on CB1 or CB2 receptors and are designed to create cannabis-like properties and reduce undesirable side effects [27,29].

On other hand, the CB1 receptor is found in the basal ganglia and cerebellum, hippocampus, and layers I and VI of the cortex, and binding to these receptors results in impaired memory and cognition; it can also be found in the vascular system, muscles, gastrointestinal tract, and reproductive organs. The CB2 receptor is found in peripheral tissues located in the marginal areas of the spleen, Peyer’s patches, and the cortex of the lymph nodes [27,29,30].

According to the literature, the main components of magistral preparations are based on THC and CBD components, and the control of certain symptoms is sought [29] (Table 2).

Due to the scarce evidence reported in the literature on the clinical use of cannabis-based magistral preparations, the objective of this review is to describe the pharmaceutical aspects and risk management focused on adverse events and interactions of the magistral preparations in three main applications: (i) management of nausea and vomiting post-chemotherapy, (ii) neuropathic pain in multiple sclerosis, and (iii) anorexia and cachexia in HIV. We also aim to show the main regulations related to these master products in Colombia.

## 2. Results

To increase consistency between the results, the three reviewers reviewed the 735 articles selected for the indication of anorexia and cachexia in HIV, 742 articles for the indication of post-chemotherapy nausea and vomiting, and 763 articles for the indication of neuropathic pain in multiple sclerosis. A free web application, Rayyan, was used as an aid in conducting systematic reviews, in which the analysis of the searches and elimination of duplicates was carried out according to each indication. Disagreements in the selection of the studies, and the extraction of the data by consensus and discussion with the other reviewers, if necessary, was documented in PRISMA format for panoramic reviews [33,34,35].

Data were extracted based on the characteristics of the article (author, year, journal, type of study, objective, type of magistral preparations used and comparator, number of patients, reported outcome, and adverse events). The studies were grouped and summarized for each of the proposed clinical indications and the type of population and study design, in addition to key measures of interest to our search. After selecting the included articles for each indication, the studies described in Table 3 were found with the key descriptions previously described. The key findings of each study can be found in Table 3.

The results obtained from the search of the literature, in general, draw attention to the small number of studies that met the inclusion criteria and the scant evidence of the use of these magistral preparations based on medicinal cannabis. Regarding the selected indications and the use of magistral preparations: anorexia and cachexia in HIV have one published article; post-chemotherapy nausea and vomiting have three articles; and for neuropathic pain in multiple sclerosis, no related article was found.

## 3. Discussion

In the studies that met the selection and inclusion criteria for the indication of post-chemotherapy nausea and vomiting, it is observed that the evidence corresponds to publications over 30 years. This indicates that there is no new evidence related to the use of these magistral preparations. Furthermore, according to the chronological order of the studies in the case of the indication of post-chemotherapy nausea and vomiting, it is observed that in the first study, by Frytak et al. in 1979, the doses of the magistral preparations based on THC were high compared to those in other studies. No maximum dose of THC was applied, and the study therefore showed a greater number of adverse events and treatment discontinuations [39].

The articles identified during the panoramic review of the characteristics of the cannabis-based magistral preparations for the two indications of anorexia and cachexia in HIV and post-chemotherapy nausea and vomiting agree with the finding in the literature that the main component of these formulas must be THC. However, it has been shown that the use of formulas rich in THC can predispose patients to an increase in psychoactive adverse events, which could be reduced with doses of CBD, given its properties of control over THC-induced psychoactive effects [27].

Regarding the excipient, only one of the magistral preparations contains sesame oil, which influences the pharmacokinetics of medicinal cannabis because it allows optimization of its absorption and bioavailability. Most of the magistral preparations based on cannabis have poor bioavailability [38]. The literature suggests the dose should start at a low level and slowly rise until the expected control of the symptoms or the presence of adverse events is achieved. The review articles note cases in which dosages are based on weight, in addition to high doses that triggered more adverse events and discontinuation rates [3,5].

However, one of the limitations described in the studies is that these medicinal cannabis-based drugs are not front-line for the treatment of established indications, but must be considered in indications that are refractory to conventional treatment, and additionally, they are not exempt from the presentation of adverse events.

Regarding the risk management of cannabis-based magistral preparations, adverse events have been recorded in relation to the use of formulas rich in THC, such as psychoactive symptoms, sedation, and alterations in mood. These are observed in greater quantity with high doses of THC compared with few adverse events associated with the comparators used (prochlorperazine and placebo), consistent with the effect being mediated by dose-dependent THC [27]. Currently, it is known that cannabis-based drugs have a safety profile compared to other drugs, with no reports of mortality or overdose due to the lack of CB1 receptors at the level of the cardiorespiratory centers of the spinal cord. In addition, the technique of “start slow and go slow” and CBD-containing preparations help mitigate adverse events [27,40]. The recommendation is to educate the patient about the most common adverse events that may occur, notify them through a pharmacovigilance program, and evaluate the dose of the magistral preparations, in addition to the patient’s susceptibility, to avoid undesirable adverse events [40,41].

In the case of drug interactions, in the indication of post-chemotherapy nausea and vomiting, Orr et al. in 1981 observed that cannabis may be more effective in controlling vomiting associated with some types of chemotherapy, such as cyclophosphamide, 5-fluorouracil, and doxorubicin, and less effective for nitrogen mustards and nitrosurea [38]. Interactions with other pharmacological groups are not documented in the articles analyzed, which indicates the need to analyze the comprehensive context of the patient and evaluate concomitant medications.

The panoramic review had a number of limitations. Some of the obtained studies did not describe the specific characteristics of the magistral preparations used, which precludes a more in-depth analysis of these magistral preparations and their clinical use in relation to the outcomes or adverse events. In addition, since the initial study found in the literature, a lack of previous studies or reports of the use of these medicinal cannabis magistral preparations has been documented, which may result in little knowledge of these magistral preparations.

The following aspects are recommended for the evaluation and management of patients who could benefit from the use of cannabis-based magistral preparations [27,42]. The clinical history should be known and an appropriate physical examination should be undertaken to evaluate the symptoms to be treated, identify diagnoses, and determine the current optimal treatment for the patient for their indication. Psychological factors and risk of substance abuse should be evaluated, and if they exist, documented. Similarly, the previous use of cannabinoids must be determined. Psychoactive drug screening should be considered and objectives for the use of these magistral preparations should be set with the patient (for example, reduction of pain, increased functionality, improved sleep and quality of life, and reduction of the use of other medications). Thus, the most appropriate treatment plan for the patient should be determined. Finally, the main adverse events, risk of addiction, monitoring, and appropriate use of cannabis-based drugs should be discussed, and a good patient–doctor follow-up relationship should be maintained.

## 4. Materials and Methods

### 4.1. Search Eligibility Criteria

The study protocol was performed using the Preferred Reporting Items for Systematic Review and Meta-Analysis Protocols (PRISMA-P) 2015 statement, in addition to the panoramic review recommendations of Levac et al. and The Johanna Briggs Institute, which have been reviewed in detail [33,34,35,43,44].

To be included in the review, the articles had to be original articles, literature reviews, narrative reviews, systematic reviews, or meta-analyzes that examined the use of magistral preparations of medicinal cannabis for the management of post-chemotherapy nausea and vomiting, neuropathic pain in multiple sclerosis, and anorexia and cachexia in patients with HIV, in a population of men and women over 18 years of age. No restrictions on publication time or language were applied. Each indication was searched for separately. The articles were excluded if they did not fit with the conceptual framework of the study or focus on the use of the magistral preparations in the three previously established indications. Letters to the editor and editorial comments were also excluded.

### 4.2. Search Strategy, Study Selection, and Data Extraction

**Figure 1:**
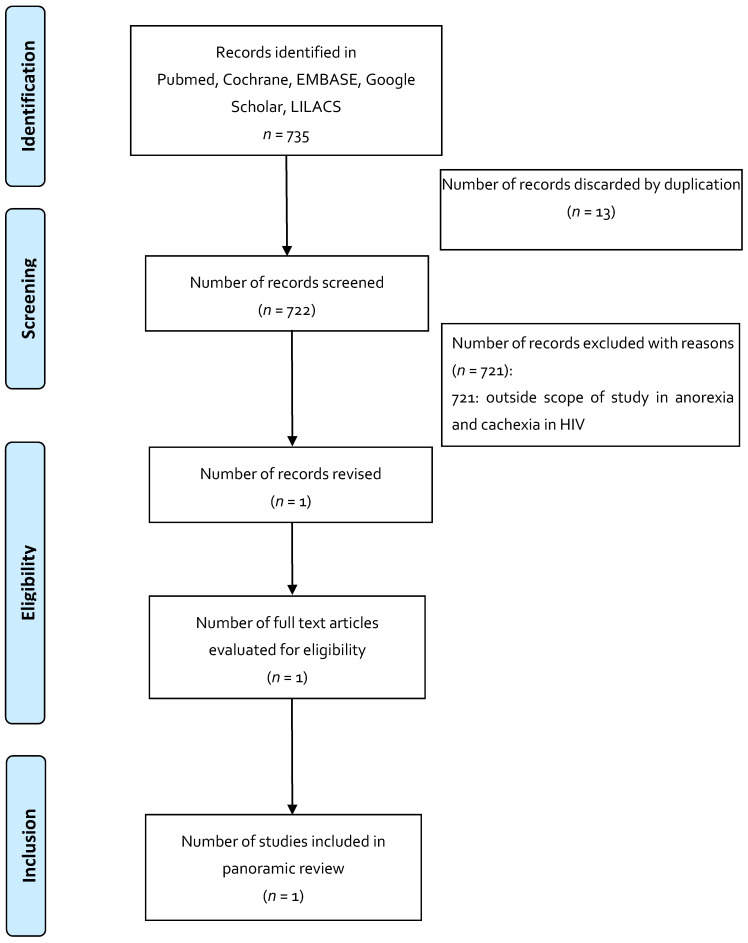
Flowchart depicting the selection process for the studies included in the paper: indication in anorexia and cachexia in HIV.

**Figure 2:**
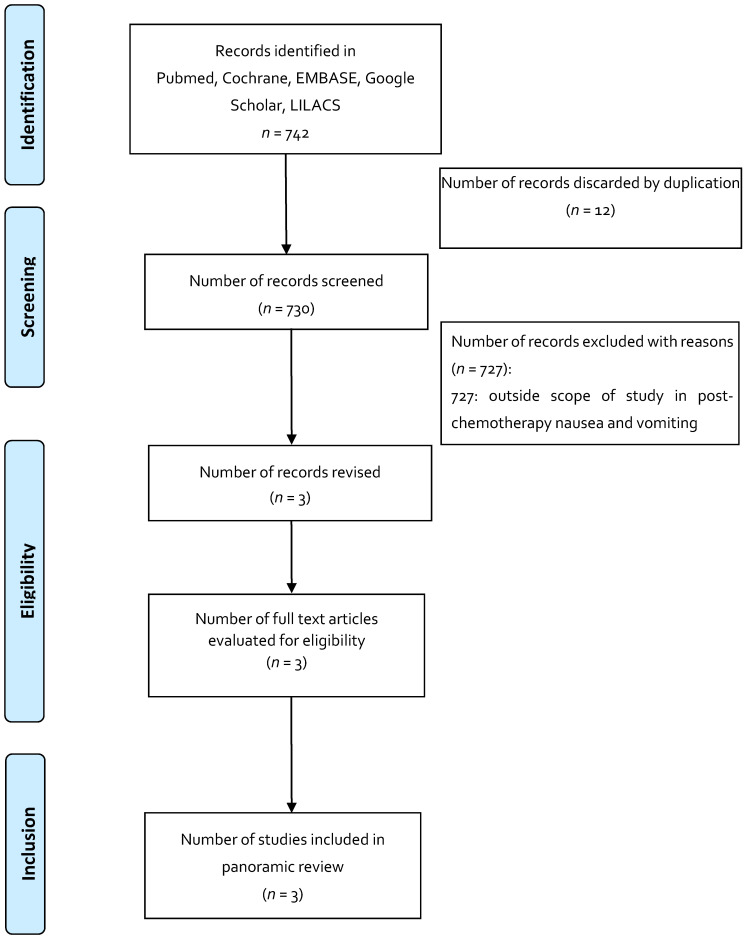
Flowchart depicting the selection process for the studies included in the paper: indication in nausea and vomiting after chemotherapy.

**Figure 3:**
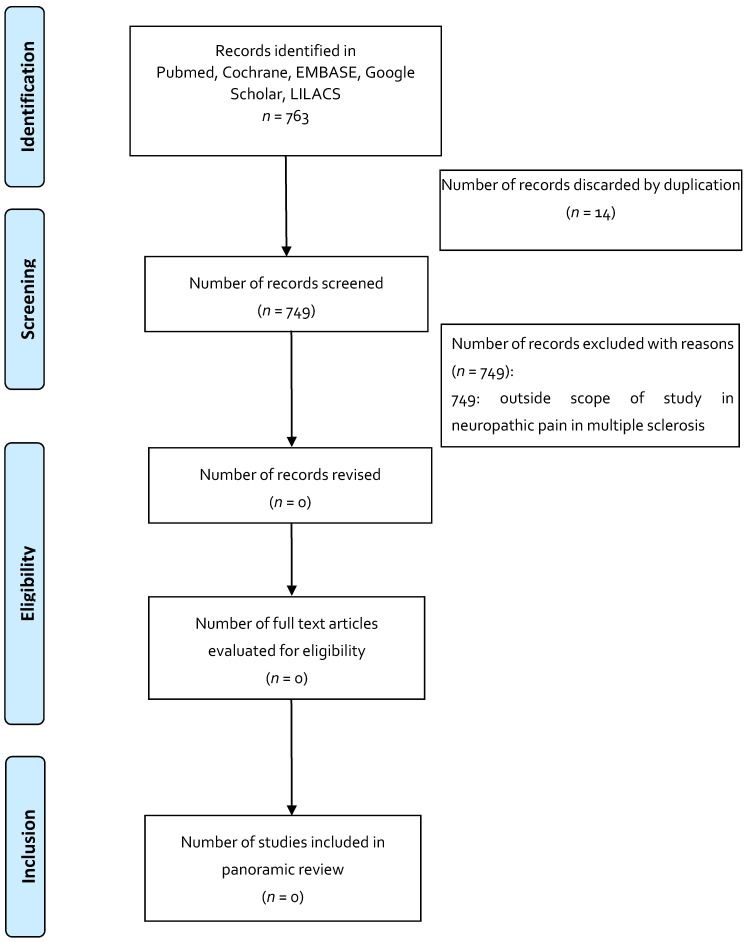
Flowchart depicting the selection process for the studies included in the paper: indication in neuropathic pain in multiple sclerosis.

Published articles (published until April 2020) that reported the use of magistral preparations were identified using computerized literature searches in Pubmed, Medline, EMBASE, Google Scholar, Cochrane, LILACS, and OpenGrey (Table 4). The search included the main symposia and congresses on medicinal cannabis. The selection of articles was made by two independent researchers, and discrepancies were resolved by consensus. The search strategy is presented in Figure 1, Figure 2 and Figure 3.

## 5. Conclusions

Magistral preparations based on medical cannabis are specific preparations for a patient who is undergoing optimal treatment, or in the case of refractory conditions, because there is not enough evidence to indicate the efficacy of these preparations as a front-line treatment. These preparations require a prescription from a doctor who determines the potential benefit of their use. Although it is known that cannabis-based drugs have a favorable safety profile compared to other drugs, there is not enough conclusive evidence in this regard. This research provides key tools to understand the importance of individualizing prescriptions for these types of formulas, monitoring them in the context of efficacy and safety, and monitoring the therapeutic levels for their use in the examined indications.

## Figures and Tables

**Table 1:** Key aspects of the pharmacokinetics of medicinal cannabis [26].

Pharmacokinetics	Characteristics
Absorption	The main forms of administration and formulations are: - Smoke or vapor inhalation: herbal cannabis, resins, and concentrates - Oral: nabiximols, edibles, and tinctures - Oro-mucosal or sublingual: lollipops, pills, and nabiximols - Topical or rectal: herbal cannabis, resins, and concentrates Bioavailability: around 13% after oral administration due to the high first metabolism effect. There is little relationship between the plasma concentrations of tetrahydrocannabinol (THC) and its neuropsychological effects, due to the multicompartmental pharmacokinetic pattern, with a distribution phase and rapid fall in plasma concentrations, for which the concentration/time curves indicate that the peak of maximum action occurs 7 s after administration [27,28]
Distribution	Δ-9-THC: the most psychoactive component of cannabis, 95–99% bound to plasma proteins with a volume of distribution (Vd) of 2.5–3 L. It is rapidly distributed from plasma to highly vascular tissues. Only 1% of THC crosses the blood–brain barrier, after the initial phase of distribution. THC is redistributed to less vascular tissues and accumulates in adipose tissue. Only 3% of THC circulates freely in plasma [27]. Cannabidiol (CBD): Vd is 30 L/kg [28].
Metabolism	THC: Metabolized in the liver through CYP2C9 and CYP3A4, 2C19 and 1A to 11-hydroxy-tetrahydrocannabinol (11-OH-THC), the more potent metabolite found in higher concentrations in the brain than THC. Hepatic hydroxylation of Δ-9-THC generates the compound 11-hydroxy-Δ-9-THC with a prolonged psychoactive effect [28]. CBD: Cannabidiol is metabolized to 7-OH-cannabidiol, which inhibits the metabolism of Δ-9-THC towards 11-hydroxy-Δ-9-THC and attenuates its psychoactive effects, in such a way that this additional oxidation renders 11-Nor-9-carboxy-Δ-9-tetrahydrocannabinol inactive, giving it greater security [27].
Elimination	It is eliminated unchanged and as a metabolite in urine (16%) and feces with an average of t ½ of 24 h [28].

Vd: volume of distribution, L: liter, kg: kilogram.

**Table 2:** Main effects of each magistral preparation based on THC and CBD.

Magistral Preparations with Main Component of THC	Magistral Preparations with Main Component of CBD
- THC produces psychoactive effects. It has been seen that according to its properties, it can help with the control of nausea and vomiting, increase in appetite in anorexia, insomnia, and chronic pain [29].	- CBD improves the tolerability and safety of formulas and is responsible for reducing the psychoactive effects of THC. - According to its properties, it is described as a muscle relaxant and seizure controller, and has anxiolytic, anti-inflammatory, and antioxidant properties [29,31], as well as epilepsy-treating properties [32].

**Table 3:** Description of selected articles.

Study Type	Objective	Type of Magistral Preparations Used and Comparator	*n*	Outcome	Adverse Event	Conclusions	Ref.
Indication 1: Anorexia and cachexia in HIV							
Systematic Review	To assess whether cannabis (in its natural or artificial form), whether smoked or ingested, reduces morbidity or mortality in HIV-infected patients.	Cannabis smoked or ingested, THC ingested (dronabinol or any form produced). Compared to placebo, no medication, another form of cannabis.	7 studies	Primary: Mortality (HIV-related, all causes) Secondary: Subjective and objective appetite experiences: 1. Cannabis vs placebo: Weight gain. Study number: 1, participants 88 OR (95% CI) 2.40 [0.70, 8.23] 2. Changes in appetite, food, and calorie intake: insufficient data do not allow for analysis of this measure.	1 study: 1 subject discontinued the study for psychosis; 2 study: 2 discontinued the study due to sedation and changes in mood.	Evidence on the efficacy and safety of cannabis and cannabinoids is lacking; most of the studies that were analyzed were of short duration, with a small number of patients, and focused on short-term efficacy measures; one of the most important limitations is not having long-term safety data.	[36]
Indication 2: Post-chemotherapy nausea and vomiting							
RCT crossover	To determine formulation preference for THC vs Compazine.	THC (offered by NIDA and administered per body surface area as follows: 7.5 mg for 1.4 m^2^, 10 mg for 1.4–1.8 m^2^, 12.4 mg for >1.8 m^2^ vs. Compazine (10 mg prochlorperazine dose).	139 (variety of neoplasms and different QT regimens)	Reported preference: 49% for THC, 43% for Compazine, 8% no preference. Nausea reduction: There was a reduction in both groups.	Sedation and psychoactive effects were greater in the THC group.	The preference for THC was associated with more adverse events related to the magisterial preparation compared to Compazine. Most studies with THC exclude patients with emotional instability; however, it is a condition that is associated with cancer and that can interfere with the proper use of THC.	[37]
RCT, double blind, randomized, crossover	To determine the degree of effectiveness of the oral THC formula in preventing nausea and vomiting associated with QT compared with the conventional antiemetic and placebo.	Oral THC at 7 mg/m^2^ every 4 h for 4 doses (NIDA), administered in 0.12 mL of sesame oil and supplied in gelatinous capsules; oral prochlorperazine at 7 mg/m^2^ every 4 h for 4 doses and placebo (administered 1 h prior to QT).	55	Absence of nausea in 40 of 55 patients receiving THC, 8 of 55 receiving prochlorperazine, and 5 of 55 receiving placebo.	THC group AEs were euphoria, sedation, and temporary loss of physical control; AEs in the chlorperazine group were excessive sleepiness and autonomic symptoms.	It concludes that the magistral preparations with THC seem to be more effective in controlling vomiting associated with cyclophosphamide, 5-fluorouracil, and doxorubicin, and less for nitrogen mustards and nitrosurea.	[38]
RCT, double blind, randomized, crossover	To observe the effect of THC as an antiemetic agent in cancer patients and to compare the antiemetic and AE effect of THC with those using prochlorpromazine.	THC: 15 mg orally 3 times per day vs. prochlorperazine 10 mg orally 3 times per day or placebo.	116	Occurrence of no nausea and vomiting events on days 2–4 (mild emetic stimulus): THC (57), prochlorperazine (72), placebo (53).	Greater degree of intolerable sedation in the THC group (5) vs. chlorperazine (2). 12 patients from the THC group (1 from pbo, 1 from chlorperazine) discontinued the study due to central intolerable symptoms.	Concludes that the THC-rich magistral preparations had superior antiemetic activity compared to placebo, but had no advantages compared to prochlorperazine. Adverse events related to the central nervous system were more frequent and severe with THC, with the dose and scheme used in the elderly population; additionally, the use of the formula rich in THC resulted in unpleasant experiences compared to prochlorperazine and placebo. Describes that the formula used shows that it prevents post-chemotherapy nausea and vomiting, yet its role must be clarified before it is recommended for general use.	[39]
Indication 3: Neuropathic pain in multiple sclerosis							
None							

RCT: randomized clinical trial, AE: adverse events, CI: confidence interval, NIDA: National Institute on Drug Abuse, n: number, QT: chemotherapy, THC: tetrahydrocannabinol, HIV: human immunodeficiency virus.

**Table 4:** MeSH and DeSC used in electronic databases.

TERMS	MeSH	DeSC
Anorexia and cachexia in HIV	Anorexia AND HIV	anorexia AND HIV
Medicinal cannabis	Medical Marijuana	medical cannabis
Magistral preparations	Pharmaceutical Preparations	pharmaceutical preparations
Post-chemotherapy nausea and vomiting	Nausea AND Vomiting AND Chemotherapy	Nausea AND Vomiting AND Chemotherapy
Neuropathic pain in patients with multiple sclerosis	Chronic Pain OR Multiple Sclerosis	Chronic Pain OR Multiple Sclerosis
